# Effects of tongue cleaning on bacterial flora in tongue coating and dental plaque: a crossover study

**DOI:** 10.1186/1472-6831-14-4

**Published:** 2014-01-14

**Authors:** Miki Matsui, Naoyuki Chosa, Yu Shimoyama, Kentaro Minami, Shigenobu Kimura, Mitsuo Kishi

**Affiliations:** 1 Division of Preventive Dentistry, Department of Oral Medicine, Iwate Medical University School of Dentistry, 3-27 Chuodori 1-chome, Morioka 020-8505, Japan; 2 Division of Cellular Biosignal Sciences, Department of Biochemistry, Iwate Medical University Basic Medicine, 2-1-1 Nishitokuta, Yahaba-cho, Shiwagun, Morioka 028-3694, Japan; 3 Division of Molecular Microbiology, Department of Microbiology, Iwate Medical University Basic Medicine, 2-1-1 Nishitokuta, Yahaba-cho, Shiwagun, Morioka 028-3694, Japan

## Abstract

**Background:**

The effects of tongue cleaning on reconstruction of bacterial flora in dental plaque and tongue coating itself are obscure. We assessed changes in the amounts of total bacteria as well as *Fusobacterium nucleatum* in tongue coating and dental plaque specimens obtained with and without tongue cleaning.

**Methods:**

We conducted a randomized examiner-blind crossover study using 30 volunteers (average 23.7 ± 3.2 years old) without periodontitis. After dividing randomly into 2 groups, 1 group was instructed to clean the tongue, while the other did not. On days 1 (baseline), 3, and 10, tongue coating and dental plaque samples were collected after recording tongue coating score (Winkel tongue coating index: WTCI). After a washout period of 3 weeks, the same examinations were performed with the subjects allocated to the alternate group. Genomic DNA was purified from the samples and applied to SYBR® Green-based real-time PCR to quantify the amounts of total bacteria and *F. nucleatum*.

**Results:**

After 3 days, the WTCI score recovered to baseline, though the amount of total bacteria in tongue coating was significantly lower as compared to the baseline. In plaque samples, the bacterial amounts on day 3 and 10 were significantly lower than the baseline with and without tongue cleaning. Principal component analysis showed that variations of bacterial amounts in the tongue coating and dental plaque samples were independent from each other. Furthermore, we found a strong association between amounts of total bacteria and *F. nucleatum* in specimens both.

**Conclusions:**

Tongue cleaning reduced the amount of bacteria in tongue coating. However, the cleaning had no obvious contribution to inhibit dental plaque formation. Furthermore, recovery of the total bacterial amount induced an increase in *F. nucleatum* in both tongue coating and dental plaque. Thus, it is recommended that tongue cleaning and tooth brushing should both be performed for promoting oral health.

## Background

The tongue dorsum occupying huge area of oral mucosa is able to harbor microorganisms including periodontopathic bacteria in addition to oral streptococci [1-4]. Furthermore, tongue mucosa is a major habitat of *Candida* species, which can cause severe infections in immunocompromised hosts such as patients in the perioperative period or bedridden elderly [5]. Such microorganisms aggregate with mucosal epithelium detachment, as well as food and saliva components, and others, and cover the tongue surface to form the so-called tongue coating. It has been reported that detection rates of periodontopathic bacteria in tongue coating were closely associated with those in dental plaque [6] and periodontal conditions [4,7-9]. Furthermore, following the loss of all natural teeth, there is a decreased prevalence of selective periodontopathic bacteria on the tongue [8,10,11]. In addition, during periods of refraining from oral hygiene, periodontopathic bacteria in the tongue coating increase along with the accumulation [12]. Based on those findings, it is considered that tongue coating and dental plaque have a reservoir and acceptor relationship to share oral microorganisms, and likely that tongue cleaning has some effect on plaque formation. However, studies that investigated tongue cleaning for the purpose of reducing formation of dental plaque have reported conflicting results. Gross, et al., observed a reduction in amount of plaque adhesion after tongue cleaning [13], whereas Badersten, et al., reported that tongue cleaning did not inhibit plaque formation [14]. Also, other studies that used culture methods found a slight or no decrease in bacterial load even on the tongue dorsum, when the degree of tongue coating was reduced [15-17]. Therefore, tongue cleaning is rarely recommended by dental professionals for oral health of common individuals except for prevention of oral malodor [18-20]. In the present study, we utilized a crossover design and compared changes in total bacteria amounts in dental plaque and tongue coating samples obtained from subjects with and without tongue cleaning using polymerase chain reaction (PCR) assays.

Previous studies have reported a relationship between periodontopathogens in tongue coating and periodontal conditions [7-9], suggesting that periodontopathic organisms in the tongue coating as well as dental plaque are an important factor in the etiology of periodontal diseases. *Porphyromonas gingivalis*, *Treponema denticola*, and *Tannerella forsythia*, known as the red complex, are believed to be prominent periodontopathic bacteria. These species are rarely detected in dental plaque or oral mucosa from individuals without periodontitis [4,7,9]. As compared to those, *Fusobacterium nucleatum*, which has also been implicated in the etiology of periodontal diseases, is frequently isolated from tongue coating and dental plaque samples regardless of periodontal condition [4,21,22]. This species represents a bridge between early and late colonizers in dental plaque, since it can co-aggregate with various oral bacteria including red complex species [23-25]. It was also reported that *F. nucleatum* growth is dependent on an increase in plaque thickness yielding anaerobic condition [26]. Furthermore, *F. nucleatum* under oxygenated and CO_2_-depleted environments supports the growth of *P. gingivalis,* thus it is possible that its colonization triggers periodontopathic bacterial colonization [27,28]. Accordingly, it is considered that the amount of *F. nucleatum* can be used to represent the microbial etiology of dental plaque and tongue coating for periodontal diseases in individuals without periodontitis. In the present study, we assessed etiological shifts in addition to quantitative changes in tongue coating and dental plaque under re-construction by determining the amount of *F. nucleatum* in collected specimens as well as total bacteria amount, and examined the relationship between those amounts.

## Methods

### Subjects

The subjects were 30 systemic healthy volunteers (mean age 23.7 ± 3.2 years, range 20–34 years) without clinical periodontitis and no missing teeth who were not undergoing antibiotic or other antimicrobial therapy within 3 months prior to the examination. They received verbal and written information about the study, and signed consent forms prior to participation. The study protocol was approved by the Ethics Committees of Iwate Medical University School of Dentistry (#01140).

### Study design

This study was a randomized, examiner blind and crossover design with a 3 weeks washout period between the crossover phases. In the baseline of first test phase, tongue coating deposits in all subjects were visually assessed. After collecting tongue coating and dental plaque samples, the subjects were randomly divided into 2 groups. One group was instructed to mechanically clean their tongues with a disposable tongue cleaner equipped with a cleaner head composed of a urethane sponge covered with a nonwoven fabric (Tongue Clean®, JCB Industry Limited, Japan) until the examiner visually confirmed that the tongue coating was completely removed. The other group performed no tongue cleaning. All of the subjects continued their habitual oral hygiene and were instructed to not clean their tongues by any means during this phase of the test period. Three and 10 days later, tongue coating assessments and collection of tongue coating and dental plaque samples were performed in the same manner as for the baseline examination. Tongue coating assessments and sample collections were done at the same time on each examination day (between approximately 16:00 and 17:00). Next, a washout period was conducted for 3 weeks, during which the subjects performed their normal oral hygiene without tongue cleaning. After the washout period, the subjects were allocated to the alternate group and the protocol was repeated. Tongue coating assessments and sampling of oral specimens were performed by the same single examiner respectively throughout the study, who was unaware of the group allocation of the subjects.

### Tongue coating assessment

Tongue coating was assessed using the Winkel tongue coating index (WTCI) [29]. Briefly, the dorsum of the tongue was divided into 6 areas (3 posterior, 3 anterior) and tongue coating was assessed in each sextant as follows; 0 = no coating, 1 = light coating, 2 = severe coating. The WTCI was obtained by adding all 6 scores, for a possible range of 0–12.

### Tongue coating and dental plaque sampling

After removing saliva from the tongue dorsum with cotton and a stream of air, any tongue accretion between the lingual papillae was carefully removed using 3 scratching strokes (approximately 1 cm long) with a sterile micro-spatula from the posterior-center area of the tongue dorsum. On day 3, tongue coating was collected in a similar manner from the right or left side (randomly chosen) a distance of 0.5 cm from the sampling area used for the baseline. On day 10, tongue coating was similarly collected from the opposite side of that used on day 3. Dental plaque samples were also collected after drying with cotton using a sterile dental explorer from the entire lingual surfaces of the first molar and second premolar on both sides of the mandibular for the baseline specimens. Subsequently, plaque samples were obtained from the tooth surfaces on either side randomly selected on day 3 and from the other side on day 10. Immediately after determining wet weight using an electronic balance (AG245, Mettler Toledo, Greifensee, Switzerland), the samples were immersed in phosphate-buffered saline (PBS, pH 7.0) and washed 3 times, then frozen at -80°C for storage. Since dental plaque was collected from both sides, the wet weight and bacteria values at the baseline were estimated as the half amounts of measured values.

### DNA extraction and quantification

DNA was extracted from samples using a Wizard® Genomic DNA Purification Kit (Promega, Fitchburg, WI, USA), according to the manufacturer’s instructions for isolating genomic DNA from gram Gram-positive bacteria. Bacterial genomic DNA was dissolved in TE buffer (10 mM Tris–HCl, 1 mM EDTA, pH 8.0) and stored at 4°C.

### Quantification of species in biofilms by real-time PCR

Specific primers were used, as follows. For 16S rRNA universal, forward: TGG AGC ATG TGG TTT AAT TCG A and reverse: TGC GGG ACT TAA CCC AAC A [30], for *F. nucleatum* ATCC25586, forward: GCG GAA CTA CAA GTG TAG AGG TG and reverse: GTT CGA CCC CCA ACA CCT ACT A [31]. The annealing temperature for both was 60°C. Quantifications of universal species and *F. nucleatum* in the samples were performed by real-time PCR analysis using SYBR® Green dye to detect the 16S rRNA gene amplicons. Each reaction mixture (final volume, 20 μL) contained 1 μL (1 ng) of template, 7 μL of ultrapure water, 10 μL of SYBR® Premix Ex Taq™ II (Perfect Real Time), and 1 μL each of the forward and reverse primers (10 μM). Real-time PCR was performed with a Thermal Cycler Dice® real-time PCR system (TaKaRa, Japan) using the following thermal cycle recommended for the SYBR® Premix Ex Taq™ II mixture: 95°C for 30 seconds, then 40 cycles for 5 seconds at 95°C and 1 minute at 60°C.

Dissociation curves were generated by incubating the reaction products at 95°C for 15 seconds and at 60°C for 30 seconds, and then incrementally increasing the temperature to 95°C for 15 seconds. Fluorescence data were collected at the end of the 60°C primer annealing step for 40 amplification cycles and throughout the dissociation curve analysis. A standard curve was generated based on the known weight of genomic DNA purified from *E. coli* ATCC 53868 and *F. nucleatum* ATCC 25586. The weight of the genomic DNA for 16S rRNA universal and *F. nucleatum* were considered to reflect the amounts of total bacteria and *F. nucleatum*, respectively. From the measurements, we calculated amounts of bacteria in each collected whole sample and amounts per a certain sample (1 mg).

### Statistical analysis

All values excluding WTCI were transferred to logarithms to improve normality. The variables were applied to the following analyses after confirming normality using a one sample Kolmogorov-Smirnov test. Differences in bacterial amounts between examination days were examined using a paired t-test with Bonferroni adjustment. Pearson’s correlation analysis was used to examine the relationship between 2 variables. In addition, principal component analysis was carried out to examine the relationships among multiple measurements. Also, the amounts of total bacteria on days 3 and 10 as ratios to the baseline were compared between the groups with and without tongue cleaning using a Wilcoxon test. Statistical analyses were performed using IBM SPSS ver. 20.0, with differences considered to statistically significant at *p* <0.05.

## Results and discussion

### Baseline measurements

At baseline, there were no significant differences for WTCI, amounts of collected tongue coating and dental plaque samples, and amounts of total bacteria and *F. nucleatum* in whole collected samples as well as those in 1-mg samples between subjects with and without tongue cleaning (Table 1). Furthermore, there were no significant differences for those parameters between the first and second baseline measurements after the 3-week washout period (data not shown). These results showed that the oral inhabitants returned to baseline levels in regard to amounts and bacterial load during the washout period, indicating that 3 weeks was sufficient for this crossover study.

**Table 1:** Baseline measurements for tongue coating and dental plaque samples

	**Tongue cleaning**	**Average ± SD**	*p*-value*
WTCI	(+)	5.53 ± 4.53	0.651
	(-)	5.90 ± 3.59	
	Total	5.72 ± 4.06	
Wet weight of tongue coating (mg)	(+)	15.4 ± 10.1	0.125
	(-)	12.0 ± 8.06	
	Total	13.6 ± 9.20	
Wet weight of dental plaque (mg)	(+)	2.75 ± 7.83	0.375
	(-)	1.31 ± 1.42	
	Total	2.03 ± 5.63	
Amount of total bacteria in whole tongue coating sample (log pg)	(+)	4.76 ± 1.18	0.296
	(-)	4.52 ± 1.15	
	Total	4.64 ± 1.16	
Amount of total bacteria in whole dental plaque sample (log pg)	(+)	4.47 ± 0.79	0.986
	(-)	4.47 ± 1.10	
	Total	4.46 ± 0.95	
Amount of *F. nucleatum* in whole tongue coating sample (log pg)	(+)	2.19 ± 1.18	0.199
	(-)	1.94 ± 1.27	
	Total	2.07 ± 1.22	
Amount of *F. nucleatum* in whole dental plaque sample (log pg)	(+)	2.07 ± 1.00	0.664
	(-)	1.98 ± 1.23	
	Total	2.02 ± 1.11	
Amount of total bacteria in 1 mg of tongue coating (log pg/mg)	(+)	3.68 ± 1.02	0.536
	(-)	3.56 ± 0.96	
	Total	3.62 ± 0.98	
Amount of total bacteria in 1 mg of dental plaque (log pg/mg)	(+)	4.51 ± 0.61	0.361
	(-)	4.44 ± 0.83	
	Total	4.48 ± 0.72	
Amount of *F. nucleatum* in tongue coating sample (log pg/mg)	(+)	1.26 ± 0.93	0.668
	(-)	1.13 ± 0.99	
	Total	1.20 ± 0.96	
Amount of *F. nucleatum* in dental plaque sample (log pg/mg)	(+)	2.09 ± 0.97	0.343
	(-)	1.90 ± 1.06	
	Total	2.00 ± 1.01	

*Statistical comparisons between subjects with and without tongue cleaning were performed using a paired t test for WTCI and bacterial amounts. Comparisons of wet weights of samples were performed using a Wilcoxon test, as they were not normally distributed.

The volume of tongue coating was greater than dental plaque, while the amounts of both total bacteria and *F. nucleatum* in the 1-mg samples were greater in those from dental plaque. These findings showed that the density of total bacteria and *F. nucleatum* was higher in dental plaque than tongue coating at the baseline. In addition, *F. nucleatum* was detected in all samples of tongue coating and dental plaque collected at the baseline.

### Change in amounts of total bacteria in tongue coating and dental plaque following tongue cleaning

**Figure 1:**
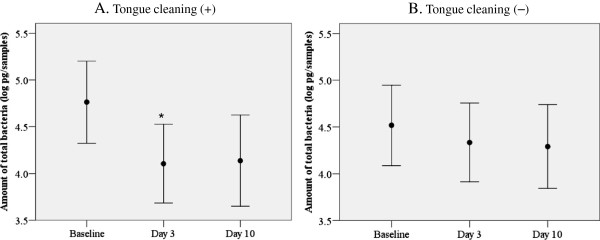
**Change in amount of total bacteria in tongue coating following tongue cleaning.** Error bars indicate 95% confidential intervals. Values shown in closed circles are averages of the amount of total bacteria expressed as logarithm values of the genome weight of 16S rRNA universal in whole collected tongue coating samples **(A)** following tongue cleaning and **(B)** without tongue cleaning. *Statistically significant, multiple paired t test with Bonferroni adjustment.

In subjects who performed tongue cleaning, the average amount of total bacteria in whole collected tongue coating samples was lower on day 3 (4.11 ± 1.13 pg, average ± SD) than at the baseline (4.76 ± 1.18 pg). Intra-group comparisons using a paired t test showed a *p*-value for the difference between day 3 and baseline of less than 0.01, which indicated a statistically significant difference in multiple comparisons of the 3 examination days after Bonferroni adjustment. A lower level of total bacteria was also observed on day 10 (4.14 ± 1.30 pg), though the difference as compared to the baseline was not significant (Figure 1A). In contrast, in subjects who did not perform tongue cleaning, the total bacterial amounts were not significantly different between the examination days (Figure 1B).

As for genome weight, the difference between baseline and day 3 in the group with tongue cleaning was 4.55 pg (actual value). Our preliminary examination showed that with *E. coli* at a genome weight of 1 ng corresponded to 3.6 × 10^4^ CFU, thus a weight of 4.55 pg was approximately equivalent to 1.9 log CFU of *E. coli*.

Quirynen et al. [16] reported that the reduction of bacterial load on the tongue dorsum after 6 months of daily tongue cleaning was less than 0.4 log CFU, which was not significant as compared to the baseline value. They also suggested that difficulty in reducing the bacterial load on the tongue is due to the surface characteristics of the tongue dorsum where innumerable depressions exist, as that structure provides ideal niches for bacterial adhesion and growth, and shelter from cleaning actions. However, Bordas et al. [17] reported significant changes in bacterial load on the tongue dorsum following 3 days of tongue scraping, with the reduction ranging from 1.11-1.96 log CFU. Our finding seems to be in agreement with the latter, though they used a cultivation method. On the other hand, when considering differences in sampling volume and frequency of tongue cleaning, the bacterial reduction by single tongue cleaning was greater and continued for a longer period than found in that previous study. Real-time PCR is able to quantify the total bacterial amount including non-cultivable bacteria with high sensitivity, whereas as much as 50% or more of the microbiota in oral biofilm have yet be successfully cultured [32,33]. Therefore, the differences between the present and previous studies may be mainly derived from different bacterial detection methods utilized.

Subsequently, for inter-group comparisons, the rates of total bacterial amounts on days 3 and 10 against the baseline were compared between subjects with and without tongue cleaning using a Wilcoxon test. There was a tendency that subjects had more for a greater reduction in bacterial load against the baseline in subjects who cleaned their tongue, though the difference was not significant (*p* = 0.106). Furthermore, there was no difference between the groups on day 10 (*p* = 0.478). Thus, the previous and present results show that the effect of tongue cleaning on reduction of bacterial amount is not remarkable, and it remains unclear whether tongue cleaning has a practical effect to reduce bacterial load in the whole oral cavity.

**Figure 2:**
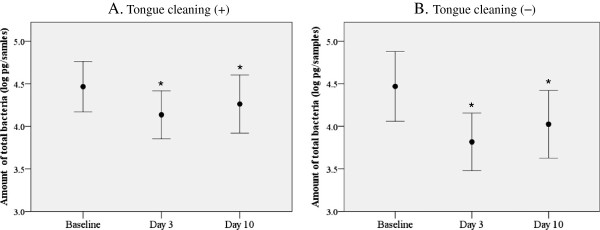
**Changes in amount of total bacteria in dental plaque after removal.** Values shown in closed circles are averages of the amount of total bacteria expressed as logarithm values of the genome weight of 16S rRNA universal in whole collected dental plaque samples **(A)** following tongue cleaning and **(B)** without tongue cleaning. Error bars and asterisks are the same as in Figure 1.

On the other hand, the average amounts of total bacteria in the whole collected dental plaque samples were significantly lower at 3 and 10 days after removal as compared to the baseline value, as shown by a paired t test with Bonfferoni adjustment (Figure 2A). This was also true in subjects without tongue cleaning (Figure 2B). Furthermore, a Wilcoxon test performed similarly to analyze tongue coating revealed there was no significant difference between the groups on either day (*p* = 0.280 on day 3, *p* = 0.380 on day 10). Together, these results suggest that tongue cleaning does not contribute to inhibition of dental plaque formation.

### Change in WTCI score after tongue cleaning

**Figure 3:**
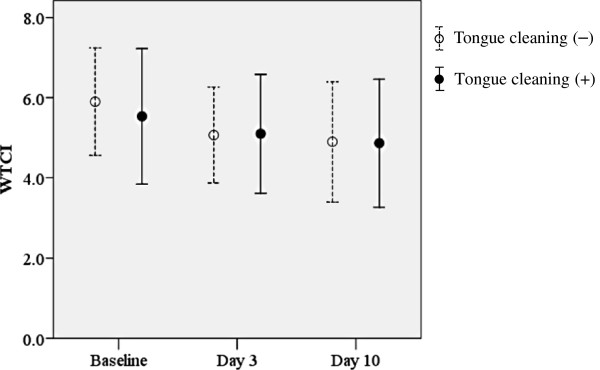
**Changes in WTCI following tongue cleaning.** Values shown in open circles are averages in subjects without tongue cleaning and those in closed circles are averages in subjects with tongue cleaning. There were no significant differences between the subject groups or examination days.

In contrast to the changing profile of total bacterial amount in tongue coating, WTCI score did not show a significant difference among the examination days in both groups (Figure 3). These findings agree with a study by Chérel, et al., who reported that average tongue coating scores returned to baseline levels 2 days after tongue cleaning [34]. Other reports have also noted disagreement between change in bacterial load on the tongue and tongue coating score after tongue cleaning [15-17]. Thus, components other than microorganisms in tongue coating are generally evaluated with an ocular inspection method. On the other hand, slight reductions in WTCI as compared to the baseline even in subjects without tongue cleaning on days 3 and 10 were noted, while a reduction in amount of total bacteria in tongue coating samples from subjects without tongue cleaning was also observed (Figure 1B). Those findings may have been related to naturally occurring inter-day changes.

### Amounts of *F. nucleatum* and total bacteria in tongue coating and dental plaque

To assess etiological shift, we examined the changes in amounts of *F. nucleatum* in tongue coating and dental plaque samples. Three days after tongue cleaning, the average amount of *F. nucleatum* in tongue coating was significantly reduced as compared with the baseline (2.19 ± 1.18 to 1.75 ± 1.29 log pg; *p* = 0.006). When tongue cleaning was not performed, there was no significant difference between day 3 and the baseline (1.94 ± 1.27 vs. 2.02 ± 1.27 log pg; *p* = 0.726). In addition, there was no difference between with and without tongue cleaning on day 10.

**Figure 4:**
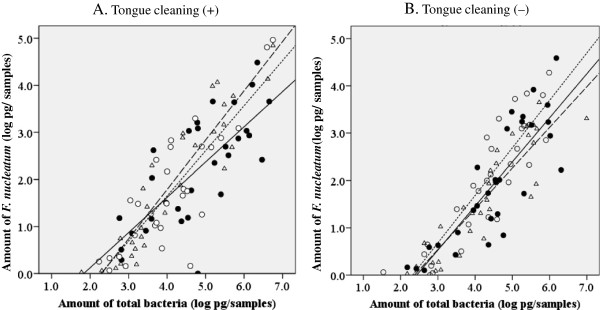
**Relationship between amounts of total bacteria and *F. nucleatum *in tongue coating.** Scatter plots of the amounts of total bacteria (X-axes) and *F. nucleatum* (Y-axes) in whole collected tongue coating samples expressed as logarithm values of the genome weight **(A)** in case with tongue cleaning and **(B)** without tongue cleaning. Closed circles, open circles, and triangles show values obtained at baseline, and days 3 and 10, respectively. Solid, dotted, and broken lines indicate approximate straight lines for baseline, and days 3 and 10, respectively. Correlation coefficients in subjects with tongue cleaning were 0.746, 0.837, and 0.928 at baseline, and on days 3 and 10, respectively, while those in subjects without tongue cleaning were 0.884, 0.844, and 0.896, respectively.

**Figure 5:**
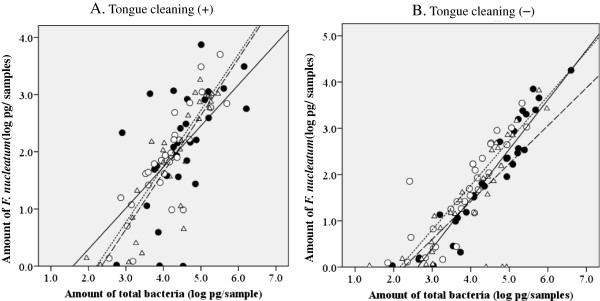
**Relationship between amounts of total bacteria and *F. nucleatum *in dental plaque.** Scatter plots of the amounts of total bacteria (X-axes) and *F. nucleatum* (Y-axes) in whole collected dental plaque samples expressed as logarithm values of the genome weight **(A)** in case with tongue cleaning and **(B)** without tongue cleaning. Symbols are the same as in Figure 4. The correlation coefficients in subjects with tongue cleaning were 0.570, 0.849, and 0.870 at baseline, and on days 3 and 10, respectively, while those in subjects without tongue cleaning were 0.952, 0.868, and 0.745, respectively.

In dental plaque, the average amount of *F. nucleatum* on day 3 was reduced after tongue cleaning but not significant (2.07 ± 1.00 to 1.88 ± 0.87 log pg; *p* = 0.145). A reduction of *F. nucleatum* on day 3 as compared to the baseline was also observed in subjects without tongue cleaning (1.98 ± 1.23 to 1.59 ± 1.00 log pg; *p* = 0.019), though the difference was not significant in multiple comparisons using Bonferroni adjustment. Since the profiles of change after removal were similar to total bacteria, we analyzed the relationship between amount of total bacteria and that of *F. nucleatum* in the samples using Pearson’s correlation analysis, and found a significant correlation coefficient. The relationship level was constantly high in both tongue coating and dental plaque with all sampling conditions used in the present protocol (Figures 4 and 5). These results showed that *F. nucleatum* occupied a certain proportion of total bacteria in both tongue coating and dental plaque during both development and under stable conditions. Furthermore, in the present study, *F. nucleatum* was detected in all tongue coating and dental plaque samples from periodontally healthy individuals, in whom the detection rates of periodontopathic bacteria such as red complex spices are often reported to be extremely low [3-6,32]. Another report noted that colonization of *F. nucleatum* induced red complex species habitation by binding both early and late colonizers in dental plaque [24]. Furthermore, we previously reported a strong correlation between dental plaque and tongue coating in regard to colonization of red complex spices [6]. Therefore, it is possible that an increase in the amount of *F. nucleatum* in tongue coating as well as dental plaque indicates an environment that is acceptable for virulent bacteria, consequently increasing the risk for periodontitis. However, we did not determine the presence of the red complex species, which is a limitation of this study.

### Overall relationships of volume and bacterial load in tongue coating and dental plaque

To review the contradictions and relationships among assessment results in the present and previous studies, we performed a principle component analysis. We applied wet weight, and amounts of total bacteria (pg/mg) and of *F. nucleatum* (pg/mg) in both tongue coating and dental plaque samples along with WTCI scores to the analysis. Table 2 summarizes the factor loadings for the measurements after Varimax rotation. The first component was strongly associated with the amounts of total bacteria and *F. nucleatum* in dental plaque, and moderately with wet weight of dental plaque. In contrast, the second component was exclusively related to amounts of total bacteria and *F. nucleatum* in tongue coating. The wet weight of tongue coating and WTCI formed another group related to the third component. These results indicated that the variations in bacterial amounts in tongue coating and dental plaque samples were largely independent of each other. Furthermore, WTCI scores were closely associated with the wet weight of tongue coating. Lundgren, et al. also found a high correlation between wet weight of tongue scrapings and WTCI [35]. On the other hand, in our study, measurements that assessed the volume of oral specimens showed a weak to moderate association with bacterial amounts among the overall variation of measurements. These results may explain the disagreement of changes after tongue cleaning between bacterial amounts and WTCI noted in our study.

**Table 2:** Component matrix after Varimax rotation following principal analysis for overall samples (n = 180)

	**Component**		
	**1**	**2**	**3**
WTCI	.187	.202	.759
Wet weight of tongue coating (log mg)	-.168	.122	.742
Amount of total bacteria in tongue coating sample (log pg/mg)	.148	.860	.301
Amount of *F. nucleatum* in tongue coating sample (log pg/mg)	.068	.957	.046
Wet weight of dental plaque (log mg)	.446	-.236	.369
Amount of total bacteria in dental plaque sample (log pg/mg)	.900	.078	-.020
Amount of *F. nucleatum* in dental plaque sample (log pg/mg)	.872	.260	-.030

Our present findings provide additional evidence to elucidate the effects of tongue cleaning, though there are some limitations. First, precise quantification using real-time PCR showed that mechanical tongue cleaning has a longer effect over time to reduce bacterial load than found in previous studies that used cultivation methods. However, it remains unclear whether the small scale reduction in bacteria observed in this study contributes to overall oral health. Second, tongue cleaning did not contribute to inhibit dental plaque formation, since the bacterial amounts in the 2 aggregates had quite different variations in an oral cavity. Finally, the volumes in tongue coating and dental plaque do not accurately represent the bacterial load in sites of attachment. In addition, the amount of *F. nucleatum* in tongue coating and dental plaque increases along with bacterial growth, which suggests an increment of virulent species in the tongue coating. These findings led us to conclude that tongue cleaning and tooth brushing should both be performed in order to reduce the amount of bacteria on the tongue and tooth surfaces, and improve the periodontal etiology.

## Conclusions

Tongue cleaning had a longer effect over time on reducing bacterial amount on the tongue as compared to ocular assessment. However, such cleaning had no obvious contribution to inhibit dental plaque formation. Thus, tongue cleaning and tooth brushing should both be performed for reducing bacterial load.

## Competing interests

The authors have no competing interest to declare.

## Authors’ contributions

MM administrated this study and was in charge of the technical procedures, including sample preparations, genome purification, and polymerase chain reaction analyses of all oral specimens, and was also responsible for writing the manuscript. NC and YS conducted microbiological analyses, and also determined the optimal conditions for detection of bacteria in the oral specimens. KM contributed to collection of clinical measurements and oral specimens. SK contributed to microbiological analysis and helped draft the manuscript. MK supervised the study and manuscript writing, and participated in performing statistical analyses. All authors have read and approved the final version of the manuscript.

## Pre-publication history

The pre-publication history for this paper can be accessed here:

http://www.biomedcentral.com/1472-6831/14/4/prepub
